# The value of neutrophil-to-lymphocyte ratio in predicting severity of coronary involvement and long-term outcome of percutaneous coronary intervention in patients with acute coronary syndrome: a systematic review and meta-analysis

**DOI:** 10.1186/s43044-024-00469-3

**Published:** 2024-03-28

**Authors:** Farzad Shahsanaei, Shahin Abbaszadeh, Soudabeh Behrooj, Nima Rahimi Petrudi, Bahareh Ramezani

**Affiliations:** Cardiovascular Research Center, Hormozgan University of Medical Sciences, Bandar Abbas, Iran

**Keywords:** Acute coronary syndrome, Major adverse cardiac event, Neutrophil-to-lymphocyte ratio, Percutaneous coronary intervention, Prognosis

## Abstract

**Background:**

The value of counting inflammatory cells and especially their counting ratio in predicting adverse clinical outcomes in patients with acute coronary syndrome (ACS) undergoing revascularization has been shown, but the results of studies have been very diverse and paradoxical. The aim of the current study was to systematically review the studies that investigated the role of increased neutrophil-to-lymphocyte ratio (NLR) in predicting long-term clinical outcomes in patients with acute coronary syndrome (ACS) undergoing percutaneous coronary intervention (PCI).

**Methods:**

Data abstraction was independently performed by both un-blinded reviewers on deeply assessing Medline, Web of Knowledge, Google Scholar, Scopus, and Cochrane Central Register of Controlled Trials and using the relevant keywords. The risk of bias for each study was assessed using the criteria outlined in the Cochrane Handbook for Systematic Reviews of Interventions and the QUADAS-2 tool. Statistical analysis was performed using the Stata software. Overall, 14 articles published between 2010 and 2021 were eligible for the final analysis.

**Results:**

A total of 20,846 ACS patients undergoing PCI were included. Higher values of NLR were associated with higher numbers of involved coronaries (RR: 1.175, 95%CI 1.021–1.353, *P* = 0.024). Increasing the value of NLR was associated with a 3.4 times increase in long-term death (RR: 3.424, 95%CI 2.325–5.025, *P* = 0.001). Similarly, higher values of NLR were significantly associated with a higher likelihood of long-term MACE (RR: 2.604, 95%CI 1.736–3.906, *P* = 0.001).

**Conclusions:**

NLR has a high value in predicting both the severity of coronary artery involvement and long-term adverse clinical outcomes following the PCI procedure.

## Background

There are multiple risk factors that can make a person more susceptible to arterial atherosclerosis. Along with genetic factors, which have been proven to play a role in various cardiovascular disorders, environmental and metabolic factors also contribute to the development of vascular disorders. Diabetes mellitus, hyperlipidemia, smoking, hypertension, and metabolic syndrome are all potential triggers for atherosclerotic processes, often by activating inflammatory processes, endothelial dysfunction, and oxidative stress [1–3]. The connection between inflammation and atherosclerosis is well-established. Inflammatory cells in the bloodstream produce cytokines and metalloproteinases, which lead to the formation of atherosclerotic plaques. These plaques increase the risk of tissue ischemia, ultimately resulting in acute myocardial infarction [4, 5]. It seems that the activation and accumulation of inflammatory cells such as neutrophils and lymphocytes also play a pathogenic role in the instability of atherosclerotic plaque [6]. It has been demonstrated that neutrophils have been recognized as key players in the process of athero-inflammation [7]. The white blood cells known as neutrophils have the ability to produce and release various enzymes, including matrix metalloproteinases, as well as different types of cytokines such as interleukin-1β and interleukin-6. These substances are believed to be the primary agents responsible for causing damage to the arterial plaque and vascular endothelial dysfunction, which in turn increases the risk of ischemia [8, 9]. In other words, neutrophil granulocytes can induce platelet aggregation in the intravascular lumen which results in increasing the extent of myocardial infarction [10]. Besides, lymphocytes have been also shown to have some inflammation-suppressing properties and therefore lowering the levels of lymphocytes may be associated with the risk for cardiac adverse sequels [11]. An increase in neutrophil count and a decrease in lymphocyte count, along with a higher ratio of neutrophils to lymphocytes, may indicate the severity of coronary vascular involvement and its long-term consequences. These adverse events are more likely to occur in patients with a history of cardiac ischemia who are undergoing revascularization procedures [12]. In this study, we thoroughly evaluated and reviewed research on the impact of a higher neutrophil-to-lymphocyte ratio (NLR) on the long-term clinical outcomes of patients with acute coronary syndrome (ACS) who are undergoing a percutaneous coronary intervention (PCI) procedure.

## Methods

### Search strategy

We conducted a study in accordance with the PRISMA-P protocols to review and analyze eligible studies on the long-term outcomes of PCI procedures. Our search involved Medline, Web of Knowledge, Google Scholar, Scopus, and Cochrane Central Register of Controlled Trials (CENTRAL) using a predefined search strategy and with the relevant keywords of “neutrophil,” “lymphocyte,” “neutrophil-to-lymphocyte ratio,” “coronary,” “percutaneous coronary intervention,” and “acute coronary syndrome.” We only included studies in English that assessed long-term outcomes of the PCI procedure and used baseline NLR to predict adverse outcomes. We included both full-text reports and abstracts that provided sufficient information for the study. The exclusion criteria were studies lacking outcome data, those in non-English languages, case reports, case series, review papers, and studies that only evaluated short-term and hospital outcomes of the procedure.

### Data abstraction and validity assessment

Both un-blinded reviewers performed data abstraction independently on structured forms. There were no differences in the data collection, but any disagreements were resolved through consensus. One of the review authors transferred the data into the Review Manager file. The data entry was double-checked by comparing it with the systematic review and extraction form. The second review author verified the accuracy of the analysis of study characteristics against the trial report. The following information was abstracted: demographic data, follow-up time, the severity of coronary valve involvement, and the number of procedural adverse events. These included long-term death and major cardiovascular adverse events (MACE), such as myocardial infarction, cerebrovascular events, renal insufficiency, thromboembolic events, or other clinical adverse events. Long-term outcomes were measured at least 12 months after PCI.

### Statistical analysis

We evaluated the risk of bias for each study based on the Cochrane Handbook for Systematic Reviews of Interventions and the QUADAS-2 tool, resolving any disagreements through discussion. Our assessment included the domains of random sequence generation, allocation concealment, blinding of participants and personnel, blinding of outcome assessment, incomplete outcome data, and selective outcome reporting, with each potential source of bias judged as high, low, or unclear. We provided quotes and justifications for our judgments in the 'Risk of bias' table and summarized the judgments for each domain across studies. We combined binary outcomes using the Mantel–Haenszel fixed effect or random-effect models. The odds ratios (ORs) and 95% confidence interval (CI) for OR were used as summary statistics for the comparison of dichotomous variables and for determining the likelihood of each adverse event after interventions. Cochrane’s Q test was used to determine the statistical heterogeneity of this study. This test was complemented with the *I*^2^ statistic, which quantifies the proportion of total variation across studies that is due to heterogeneity rather than chance. A value of *I*^2^ of 0–25% indicates insignificant heterogeneity, 26–50% low heterogeneity, 51–75% moderate heterogeneity, and 76–100% high heterogeneity. Publication bias was assessed by the rank correlation test and also confirmed by the funnel plot analysis. Reported values were two-tailed, and results were considered statistically significant at *P* = 0.05. Statistical analysis was performed using the Stata software (version 13.1, Stata Corp, College Station, TX, USA).

## Results

### Study selection and characteristics of the studies

**Fig. 1:**
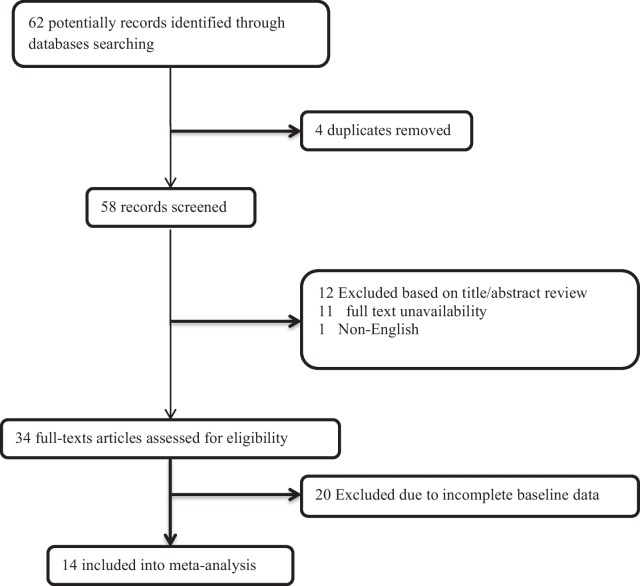
The flowchart of screening the eligible studies

The flow diagram of the study selection is presented in Fig. 1. Initially, 62 articles were collected by database searches and review of the references. After removing 4 articles due to evidence of duplication, 58 records were primarily under-screened. Based on the titles and abstracts, 24 records were excluded and the remaining 34 citations were assessed for further eligibility. Of those, 20 were also excluded due to the incompleteness of the data and contents. In total, 14 articles published between 2010 and 2021 were eligible for the final analysis [13–26] (Table 1).

**Table 1:** Details of baseline characteristics of the study population

References	NLR groups	No. population	Mean age (year)	Male (%)	Mean follow-up (month)
Shen et al. [12]	≤ 3.45	137	59.1	75.2	62
	3.45–4.81	138	59.9	73.7	
	4.82–6.46	138	61.1	75.4	
	> 6.46	138	63.9	82.6	
Han et al. [13]	≤ 3.3	108	59.7	73.1	12
	3.3–6.5	108	61.6	78.7	
	> 6.5	110	64.2	75.5	
Kaya et al. [14]	< 2.3	227	60	81	43
	2.3–4.4	228	60.9	75	
	> 4.4	227	61.7	79	
Sen et al. [15]	< 3.3	68	55.1	89.7	38
	3.3–4.5	68	56	85.2	
	> 4.5	68	56.4	83.8	
Arbel et al. [16]	< 6.5	376	60	80	87
	≥ 6.5	162	63	83	
Ergelen et al. [17]	≤ 6.97	1607	55.9	85.4	21
	> 6.97	803	57.5	82.7	
Pan et al. [18]	3	212	59.5	78.8	12
	3.0–6.4	212	59.2	76.9	
	> 6.4	212	59.1	77.8	
Zhou et al. [19]	< 2.2	350	49.06	71.1	42
	2.2–3.8	350	52.82	83.1	
	> 3.8	350	56.39	86	
Wada et al. [20]	< 1.7	729	65.9	79.6	88
	1.7–2.5	692	66.3	83.8	
	> 2.5	649	67	85.7	
Zuin et al. [21]	< 2.5	2202	64.2	74.4	12
	2.5–5.0	2262	64.5	73	
	> 5.0	2156	64.2	74.9	
Xu et al. [22]	≤ 3.3	1037	66.6	85	24
	> 3.3	396	68.2	86.4	
Choi et al. [23]	≤ 2.8	110	61.7	66.4	29
	> 2.8	104	69.2	59.6	
Liu et al. [24]	< 1.36	1139	63.3	68.8	36
	1.36–1.96	1166	63.4	70.5	
	> 1.96	1157	63.6	74.1	
Gu et al. [25]	≤ 3.3	209	65.5	67.7	40
	> 3.3	441	65	81.1	

### Methodological quality of the included studies

**Fig. 2:**
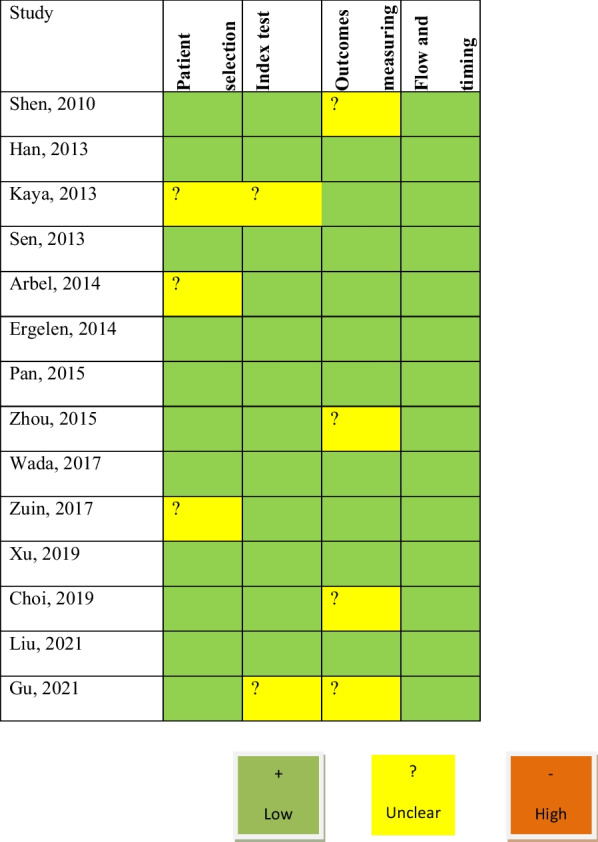
Methodological quality of the included studies

The studies included were assessed qualitatively by the QUADAS-2 tool. All 14 studies yielded good quality, and none of the citations was determined to have a high risk of bias (Fig. 2).

### Overall study characteristics

A total of 20,846 ACS patients undergoing PCI were included. Their mean age was 61.33 ± 4.39 years, range 49–69 years and 78.0% (ranged 59.6–89.7%) were male. The patients were followed up for 12–88 months for a mean follow-up time of 39 months (Table 1). With regard to coronary artery states, a wide range of multi-vessel involvement has been described in 14.4–78.8% of patients suffering from multi-vessel coronary disease (Table 2).

**Table 2:** Cardiovascular status and long-term clinical outcomes

References	NLR groups	Multi-vessel	Death	MACE
Shen et al. [12]	≤ 3.45	70.8	5.8	
	3.45–4.81	61.5	8	
	4.82–6.46	65.7	8.7	
	> 6.46	76.9	26.8	
Han et al. [13]	≤ 3.3	–	2.8	3.7
	3.3–6.5		10.2	11.1
	> 6.5		18.2	19.1
Kaya et al. [14]	< 2.3	33	8	13
	2.3–4.4	44	10	21
	> 4.4	48	18	40
Sen et al. [15]	< 3.3	26.4	9	21
	3.3–4.5	29.4	15	34
	> 4.5	32.3	35	50
Arbel et al. [16]	< 6.5	56	4.5	8.7
	≥ 6.5	49	12.9	21.1
Ergelen et al. [17]	≤ 6.97	56.8	4.8	23.7
	> 6.97	58.2	7	27.4
Pan et al. [18]	3	73.1	3.3	17.5
	3.0–6.4	78.8	5.7	19.2
	> 6.4	76.4	11	26.9
Zhou et al. [19]	< 2.2	63.5	2.5	13.1
	2.2–3.8	70.9	4.2	21.1
	> 3.8	73.5	8.2	44.5
Wada et al. [20]	< 1.7	56.4	5.3	
	1.7–2.5	63.3	6.3	
	> 2.5	61.8	8.8	
Zuin et al. [21]	< 2.5	16	0.8	
	2.5–5.0	14.9	1.4	
	> 5.0	14.4	7.5	
Xu et al. [22]	≤ 3.3	–	0.8	12.6
	> 3.3		5.1	21.7
Choi et al. [23]	≤ 2.8	33.6	2.7	3.8
	> 2.8	42.7	8.7	10.4
		48.1		
Liu et al. [24]	< 1.36	62.3	3	11.8
	1.36–1.96	62.6	3.3	10.2
	> 1.96	65.3	4.5	11.2
Gu et al. [25]	≤ 3.3	46.5	3.3	11.1
	> 3.3	47.2	9.2	28.9

### Outcome assessment

The two criteria were considered as the study end points including long-term death and long-term MACE. Regarding the death rate, the mean long-term mortality rate in the pointed population was estimated to be 8.2 ± 6.9% ranging from 1.4 to 35.0%. Furthermore, the long-term MACE rate was also shown to be 19.60 ± 11.52% ranged 3.7–50.0%.

### The value of NLR to predict long-term outcome

**Fig. 3:**
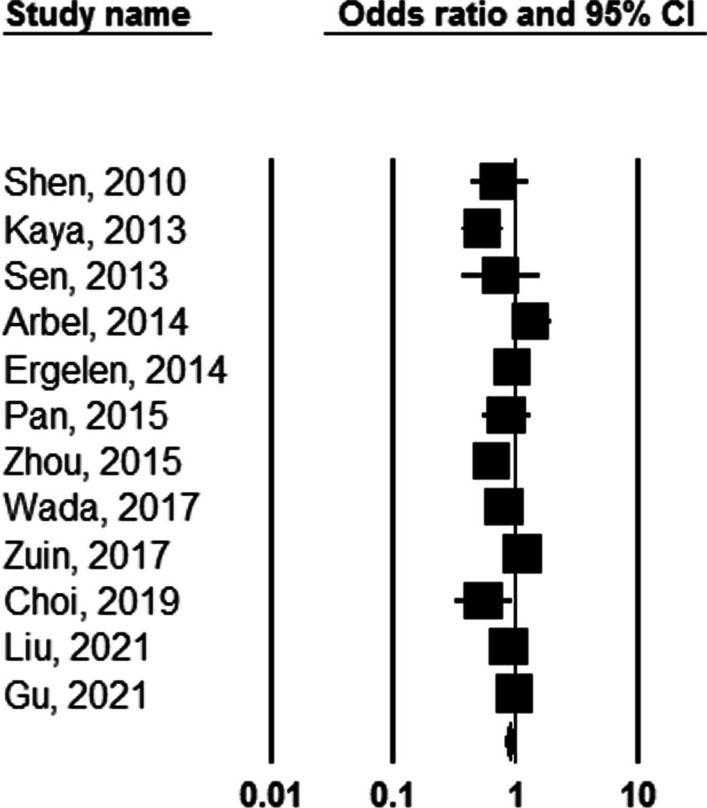
Predicting role of NLR in predicting the severity of coronary artery involvement

**Fig. 4:**
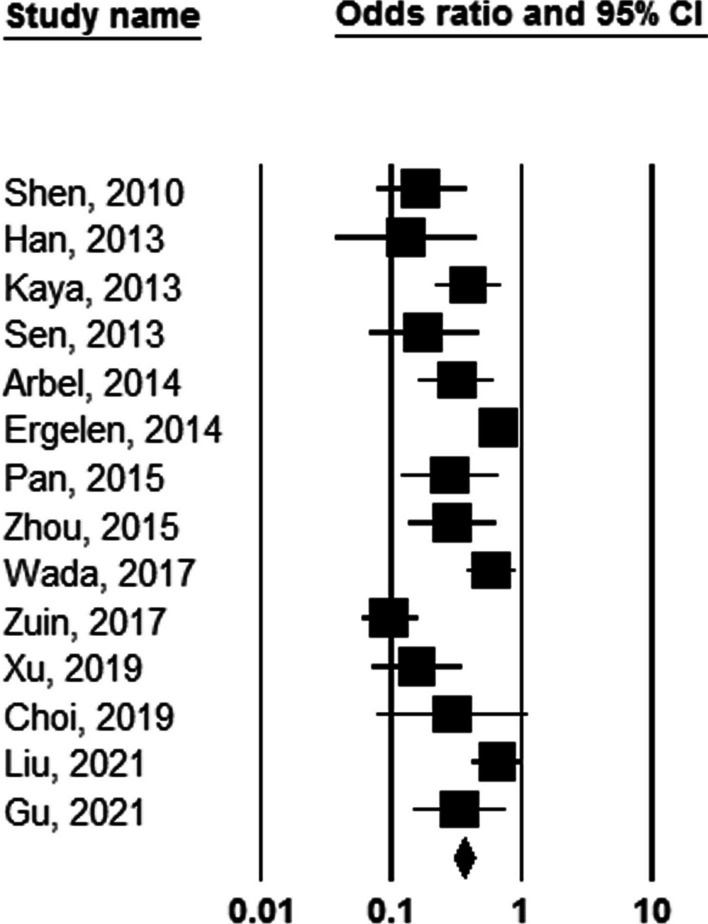
Predicting role of NLR in predicting long-term death following PCI

**Fig. 5:**
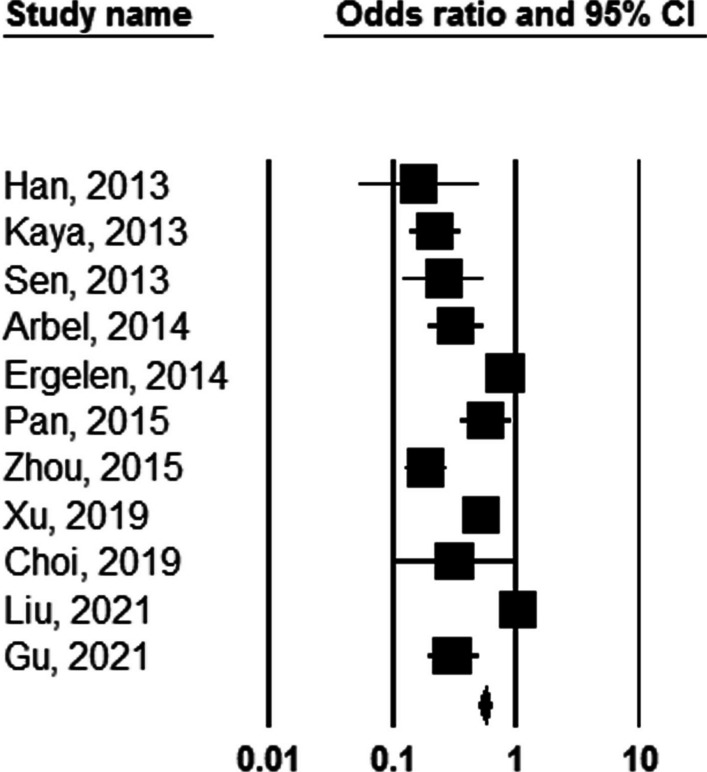
Predicting role of NLR in predicting long-term MACE following PCI

**Fig. 6:**
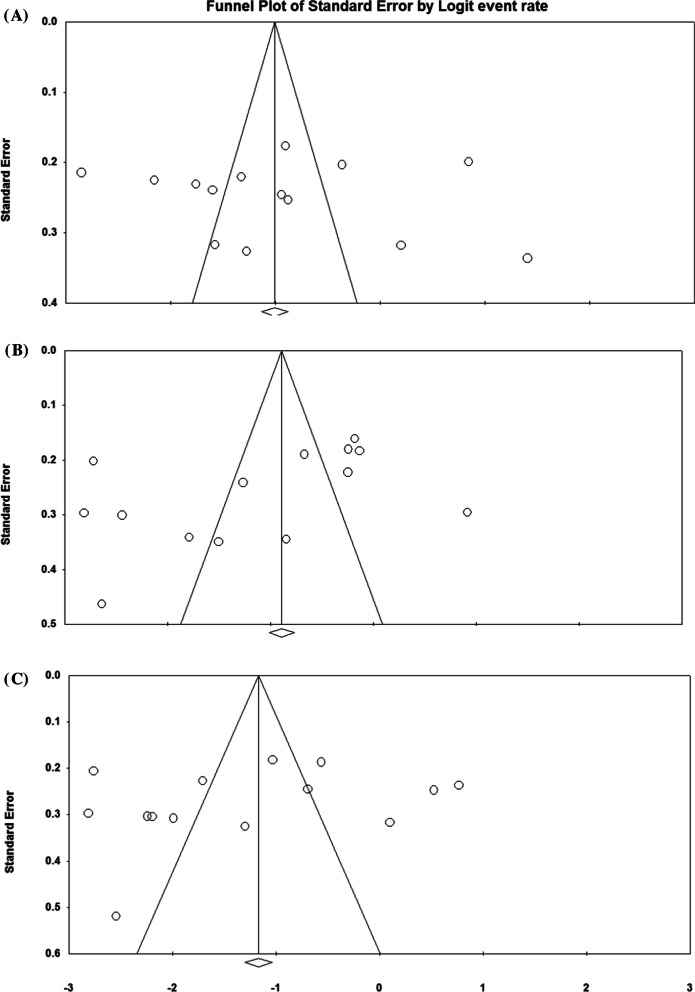
The funnel plots for assessment of the publication bias in assessment of the association of NLR with Multi-vessel (**A**), Death (**B**), and MACE (**C**)

It was first shown a significant association between NLR value and the number of involved coronary arteries that higher cutoff values of NLR were associated with higher numbers of involved coronaries (OR: 1.175, 95%CI 1.021–1.353, *P* = 0.024, Fig. 3). Besides, increasing the value of NLR (considering higher thresholds of this index) was associated with a 3.4 times increase in long-term death (OR: 3.424, 95%CI 2.325–5.025, *P* = 0.001), Fig. 4). Similarly, higher values of NLR were significantly associated with a higher likelihood of long-term MACE (OR: 2.604, 95%CI 1.736–3.906, *P* = 0.001). The heterogeneity among studies in reporting severity of coronary involvement (*I*^2^ = 62.723, *P* = 0.002), long-term death (*I*^2^ = 79.528, *P* = 0.001), and long-term MACE (*I*^2^ = 90.610, *P* = 0.001) remained significant. The publication bias is also significant for all study assessments assessed by the funnel plot with the p values ranging from 0.035 to 0.045 (Figs. 5, 6).

## Discussion

It has been fully established that inflammatory cells play a significant role in the advancement of atherosclerotic plaque formation and injury. Research has been conducted to investigate the stimulatory role of some inflammatory factors in the destruction of atherosclerotic plaque and the reparative role of other factors. The development or inhibition of atherosclerosis is the outcome of the interaction between these two types of factors [27, 28]. Research has revealed that neutrophils' secretory activity, which involves producing and secreting certain cytokines and enzymes, can contribute to atherosclerotic plaque rupture, increased platelet aggregation, and the development of atherosclerosis [29]. Meanwhile, certain secretory compounds from lymphocytes can slow down this process. As a result, it is believed that keeping track of the number of these cells can forecast the immediate and prolonged clinical results of patients. According to existing research, the NLR is highly effective at predicting the occurrence of both short-term in-hospital events and long-term outcomes in patients with ACS [30]. Studies have demonstrated that the ability to predict outcomes for patients undergoing revascularization procedures varies greatly and is often conflicting. This can be attributed to several factors, including differences in follow-up duration, varying indicators used to select patients for the study, the severity of coronary artery involvement, underlying risk factors, and the design of the study. These factors have resulted in considerable heterogeneity in our study when determining the predictive value of the NLR index for patient mortality and long-term complications. Our findings indicate that the NLR index can play a dual role. Firstly, it can predict the severity of coronary artery involvement based on the number of affected arteries. Secondly, a higher NLR threshold level is associated with increased long-term mortality rates and a higher incidence of long-term complications or MACE after the PCI procedure. In other words, increasing the mentioned ratio can increase the mortality of patients more than 3.4 times and long-term MACE more than 2.6 times. Therefore, it seems that this parameter, along with other predictive clinical and laboratory factors, can be strongly used to predict the adverse events of the procedure.

## Conclusions

It can be finally concluded that increasing NLR is associated with a higher likelihood of long-term outcomes including mortality and MACE following PCI procedure in ACS patients. Of course, the results of the studies were very heterogeneous, which could be due to the very different follow-up period as well as different definition criteria to define the long-term consequences of this procedure.

## Data Availability

All data generated or analyzed during this study are included in this published article, and further in detailed one are available from the corresponding author on reasonable request.

## Abbreviations

NLR: Neutrophil-to-lymphocyte ratio
ACS: Acute coronary syndrome
PCI: Percutaneous coronary intervention
RRs: Risk ratios
CI: Confidence interval
